# IgG Index Revisited: Diagnostic Utility and Prognostic Value in Multiple Sclerosis

**DOI:** 10.3389/fimmu.2020.01799

**Published:** 2020-08-20

**Authors:** Yang Zheng, Meng-Ting Cai, Fan Yang, Ji-Ping Zhou, Wei Fang, Chun-Hong Shen, Yin-Xi Zhang, Mei-Ping Ding

**Affiliations:** ^1^Department of Neurology, School of Medicine, Second Affiliated Hospital, Zhejiang University, Hangzhou, China; ^2^Harvard University School of Public Health, Boston, MA, United States; ^3^Department of Neurology, School of Medicine, Fourth Affiliated Hospital, Zhejiang University, Yiwu, China

**Keywords:** immunoglobulin G index, multiple sclerosis, clinically isolated syndrome, cerebrospinal fluid, McDonald criteria

## Abstract

**Objective:** Early and accurate diagnosis of multiple sclerosis (MS) remains a clinical challenge. The main objective is to evaluate the diagnostic and prognostic value of the routinely performed immunoglobulin G (IgG) index for MS patients in the Asian population.

**Methods:** A retrospective study was conducted among a cohort of clinically isolated syndrome (CIS) patients in China with known oligoclonal band (OCB) status and IgG index at baseline. We first evaluated the predictive value of IgG index for OCB status. Secondly, the diagnostic utility and prognostic value of IgG index alone were tested. Lastly, we incorporated IgG index into the 2017 McDonald criteria by replacing OCB with either “IgG index or OCB” (modified criteria 1), “IgG index and OCB” (modified criteria 2), or “IgG index” (modified criteria 3). The diagnostic utility of different criteria was calculated and compared.

**Results:** In a CIS cohort in China (*n* = 105), IgG index > 0.7 forecasted OCB positivity (*X*^2^ = 22.90, *P* < 0.001). An elevated IgG index was highly prognostic of more clinical relapses [1-year adjusted odds ratio [OR] = 1.32, *P* = 0.015; 2-years adjusted OR = 1.69, *P* = 0.013] and Expanded Disability Status Scale worsening (1-year adjusted OR = 1.76, *P* = 0.040; 2-years adjusted OR = 1.85, *P* = 0.032). Under the 2017 McDonald criteria (Positive Likelihood Ratio = 1.54, Negative Likelihood Ratio = 0.56), an IgG index > 0.7 in CIS patients increased the likelihood of developing MS within 2 years, either when OCB status was unknown (Positive Likelihood Ratio = 2.11) or with OCB positivity (Positive Likelihood Ratio = 2.11) at baseline; An IgG index ≤ 0.7, along with a negative OCB, helped rule out the MS diagnosis (Negative Likelihood Ratio = 0.53).

**Conclusions:** IgG index > 0.7 predicts OCB positivity at the initial attack of MS and is prognostic of early disease activity. IgG index serves as an easily-obtainable and accurate OCB surrogate for MS diagnosis in the Asian population.

## Introduction

Multiple sclerosis (MS) is a demyelinating disease with a poor outcome if not diagnosed and treated in time (1). Dissemination in space (DIS) and dissemination in time (DIT) constitutes the two major pillars in MS diagnosis, both of which require robust and sufficient evidence (1). For decades, correct diagnosis at the earliest time point has been the key but unresolved issue (2). Clinical manifestations alone, given its heterogeneity and subjectivity, are insufficient for this end. Paraclinical investigations, including radiological findings and cerebrospinal fluid (CSF) analysis, are therefore valuable and necessary to establish a diagnosis of MS (1).

CSF analysis, with its ability to detect intrathecally synthesized immunoglobulins, in the form of immunoglobulin G (IgG) index or CSF-specific oligoclonal band (OCB), provide critical information of central nervous system (CNS) inflammation (3, 4). However, OCB was not routinely tested for CIS patients in China, partly due to its cost and inadequate recognition among clinicians. The incomplete workup, therefore, leads to a delay in the diagnosis of MS. By contrast, IgG index, a quantitative measurement of intrathecal IgG synthesis (5), is routinely and universally performed in all the patients with suspected CIS in China (4, 6). Given its low cost and easy accessibility, IgG index is still considered a potential candidate for MS diagnosis despite a relatively low sensitivity (50–75% among MS patients) (3). Studies on its specificity, however, generated mixing results (7–10). Furthermore, the prognostic value of IgG index or OCB remains undetermined based on existing evidence (10–13).

With an extremely low prevalence of 1.39 per 100,000 among the Chinese population, the diagnosis for MS is always a challenge (14). Furthermore, MS in the Asian population may present with different clinical patterns from the Caucasian patients with less disseminated baseline MRI lesions, a more benign disease course and a lower rate of OCB positivity (21%-60% in Asians vs. 89.8% in Caucasians) (15–21). Delays in diagnosis and treatment are not uncommon. Measures aimed at facilitating an early and accurate diagnosis are greatly needed.

In this sense, in a CIS cohort in China, we aimed to examine the value of the routinely tested IgG index for MS in a way that can be implemented in future practice. We first examined the predictive value of IgG index or OCB status. Next, we evaluated the utility of IgG index alone for MS diagnosis and prognosis. Lastly, we incorporated IgG index into the 2017 McDonald criteria in different ways (“IgG index or OCB,” “IgG index and OCB„” and “IgG index alone” as evidence of DIT) and assessed their diagnostic utility, respectively.

## Methods

### Study Design and Participants

This is a retrospective study based on a prospectively collected file of patients with newly diagnosed CIS from 2012 at the Second Affiliated Hospital School of Medicine Zhejiang University, a tertiary referral hospital in Zhejiang province, China. For the purpose of this analysis, the database was locked on 31st July 2019. Inclusion criteria for the CIS cohort are (1) presentation within 3 months after an initial attack suggestive of a CNS inflammatory demyelinating event and not attributable to other diseases, with a duration of at least 24 hours; (2) age of onset between 11 to 60 years old; (3) no previous history of central nervous system demyelinating events; 4) no previous treatment with disease-modifying drugs (1). Patients eligible for inclusion in this study should have baseline IgG index and OCB data at their first clinical attack. Patients reaching an alternative diagnosis during follow-up were excluded. The demographic data, the CIS phenotype, CSF data, cranial magnetic resonance imaging (cMRI) and spinal cord MRI (sMRI) load and disability [according to the Expanded Disability Status Scale [EDSS] score] were recorded at baseline. All patients in the CIS cohort were followed up on a regular basis (every 6–12 months after onset). On follow-up, the occurrence of clinical relapses, MRI activity, the EDSS score and DMT use were recorded.

First, longitudinally, we evaluated the diagnostic value of IgG index vs. OCB alone for MS diagnosis and prognosis in the first 2 years after onset. Second, we incorporated IgG index into the 2017 McDonald criteria and assessed the diagnostic utility of the modified criteria. We modified the 2017 McDonald criteria by replacing OCB either with “IgG index or OCB” (as “modified criteria 1” below), “IgG index and OCB” (as “modified criteria 2” below) or “IgG index” (as “modified criteria 3” below).

### Outcomes

For MS diagnosis, two sets of outcomes were used. The first featured on conversion to clinically definite MS (CDMS) according to the Poser criteria, which required new symptoms suggestive of a relapse occurring after at least 1 month after CIS, and confirmed via examination (22). The second took into account conversion to McDonald MS (23). Patients were considered reaching McDonald MS when exhibiting a new T2 and/or gadolinium-enhancing lesions on follow-up MRI. Additionally, patients experiencing a second clinical attack also satisfied the McDonald MS criteria.

For MS prognosis, both disease activity and progression were evaluated at the end of 1st and 2nd year after onset. Markers for disease activity included clinical relapses and MRI activity (the total number of new T2 lesions and gadolinium-enhancing lesions on MRI) (17, 18). Disease progression was evaluated with EDSS worsening, defined as an increase of the EDSS score (24). Only EDSS evaluations performed at stable periods were considered (24). EDSS increase was confirmed at a scheduled study visit 6 months later.

### Procedures

CSF samples were collected during the first attack before steroid treatment and were analyzed for the number of white blood cells, protein and presence of elevated IgG index or OCBs. Methods for IgG index and OCB evaluations were consistent across the study period. IgG index was calculated as the CSF-plasma concentration quotient for IgG divided by the CSF-plasma concentration quotient for albumin (*Q*_IgG_/ *Q*_alb_) (5). The CSF and serum samples were measured in parallel by standard nephelometric assays. An IgG index over 0.7 was regarded as elevated (4, 6). OCB was performed using isoelectric focusing followed by immunoblotting (6, 25). OCB status was considered positive if there were ≥2 unique bands in CSF compared to serum (4, 6). Examiners were blinded to the identity and diagnosis of the patients.

Diagnostic cranial MRI scans were performed systemically at the initial visit in our hospital. Cranial MRIs were repeated every 6–12 months either at scheduled follow-up visits or at clinical visits due to relapses. Spinal MRIs were performed only when symptoms indicated spinal cord involvement. MRI examinations were acquired on a gradient echo (GE) 1.5-T MRI scanner (Siemens Healthcare, Erlangen, Germany) and included the following sequences: axial T1-weighted images (T1WIs) (400/9–14 ms, repetition time [TR]/echo time [TE]), T2-weighted images (T2WIs) (3,000–6,500/88–110 ms, TR/TE), T2-fluid-attenuated inversion recovery (FLAIR) images (7,800–9,800/100–160/1,900–2,900 ms, TR/TE/inversion time), and T1WIs with contrast enhancement (1,750–2,500/10–30 ms, TR/TE). The slice thickness ranged from 3 to 6 mm. The spinal cord MRI included axial and sagittal T2WIs (2,000–5,000/90–120 ms, TR/TE, 4 mm slice thickness), and sagittal short -tau inversion recovery sequence (2,500–3,000/70–100 ms, TR/TE, 3 mm slice thickness).

### Statistical Analysis

Descriptive statistics were performed on demographic and clinical variables. The distribution of quantitative data is described by median and range. Qualitative data are presented by absolute and relative frequencies. An estimated 91 patients would be needed for a study on the diagnostic performance of IgG index, assuming a specificity of 0.6 (based on preliminary data in our center), with a two-sided α of 0.05 and δ of 0.1 (26).

Sensitivity, specificity, positive predictive value (PPV), negative predictive value (NPV), positive likelihood ratio (PLR), negative likelihood ratio (NLR) and accuracy were calculated as described before (15). Bias-corrected and accelerated bootstrap method was used to estimate 95% confidence interval (CI). Comparison of sensitivity and specificity between IgG index and OCB was performed with the McNemar test (when b+c < 25, exact McNemar test was used).

Univariate and multivariate Cox regression analysis were used to evaluate the predictive value of IgG index/OCB/diagnostic criteria for an earlier diagnosis of MS (either CDMS or McDonald MS as the endpoint). Generalized linear multivariate regression analysis was performed using clinical relapses (linear), cMRI activity (Poisson), sMRI (binomial) and EDSS worsening (linear) as the outcome. Covariates considered for Cox analysis and generalized linear analysis included age of onset, sex, clinical topography, DIS at baseline, CSF cell count, CSF protein level and disease-modifying therapy (DMT) use prior to MS diagnosis. Covariates were only retained if they were significant in univariate analysis or if they had a substantial effect on the patient outcome.

All statistical analyses and graphs were analyzed with R (Version 3.3.3 for Mac). *P* < 0.05 were considered statistically significant and all *P* values were 2-sided.

## Results

### General Characteristics

From January 2012 to July 2019, a total of 154 patients were enrolled in the CIS cohort; 115 of them had available IgG index and OCB data at baseline; 10 were further excluded for reaching an alternative diagnosis during follow-up (neuromyelitis optica, CNS vasculitis, cerebral autosomal dominant arteriopathy with subcortical infarcts and leukoencephalopathy, hereditary leukodystrophy, CNS lymphoma and stiff person syndrome). Ultimately, 105 patients were included in this analysis. Of the 105 patients, 65 (61.9%) were female and the mean age of CIS onset was 31 [interquartile range [IQR] 25–46] years old. Eleven (10.5%) patients presented with optic neuritis, 51 (48.6%) with a spinal cord syndrome, 24 (22.9%) with brainstem symptoms, and 19 (18.1%) with other clinical features (hemispheric or multiregional) (Table 1). All 105 (100%) patients underwent cranial MRIs at baseline and follow-ups; 95 (90.5%) patients had spinal MRIs at baseline; 93 (88.6%) underwent spinal MRIs at the 1-year follow-up; 52 (49.5%) had spinal MRIs at the 2-years follow-up.

**Table 1:** General characteristics.

**DEMOGRAPHICS**	
Total, No. (%)	105 (100)
Female, No. (%)	65 (61.9)
Age at onset, median (IQR), y	31 (25–46)
**CLINICAL FEATURES**	
Phenotype, No. (%)	
Optic neuritis	11 (10.5)
Myelopathy	51 (48.6)
Brainstem/Cerebellar syndrome	24 (22.9)
Hemispheric syndrome	4 (3.8)
Polyfocal	15 (14.3)
Fulfillment of DIS at baseline	42 (40%)
Patients with cranial MRI at baseline	105 (100)
Cranial MRI load (IQR)	3.5 (2–4)
Patients with spinal MRI at baseline	95 (90.5)
Spinal MRI load (IQR)	1 (0–2)
EDSS score at baseline (IQR)	2 (2–3)
**PARACLINICAL FEATURES**	
CSF cell count (IQR)	9 (7–14)
CSF protein (IQR), mg/dl	35 (21–48)
OCB positivity, No. (%)	61 (58.1)
IgG index	0.64 (0.50–0.81)
IgG index elevation, No. (%)	44 (41.9)
**FOLLOW-UP**	
CDMS at follow-up, No. (%)	69 (65.7)
McDonlad MS at follow-up, No. (%)	80 (76.2)
Time to CDMS (IQR), m	12 (5.5–27.5)
Follow-up duration (IQR), m	32 (23–67)

*CDMS, clinically definite multiple sclerosis; CSF, cerebrospinal fluid; DIS, dissemination in space; EDSS, Expanded Disability Status Scale; IgG, immunoglobulin G; IQR, interquartile range; MRI, magnetic resonance imaging; OCB, oligoclonal band*.

Patients were followed up for a median of 32 months (IQR 23–67 months). During follow-up, 69 patients (65.7%) underwent CDMS conversion, and 80 (76.2%) reached the diagnosis of McDonald MS. Twenty (19%) patients were on immunosuppressive agents at least once (lasting at least 6 months) during follow-up, including one of the available DMTs in China (beta-interferon or teriflunomide), or off-label drugs (azathioprine, mycophenolate mofetil, or rituximab); two (1.9%) patients had initiated treatment prior to their second-attack (both on teriflunomide) and 1 (1.0%) (teriflunomide) prior to diagnosis of McDonald MS.

### Baseline CSF Characteristics

**Figure 1:**
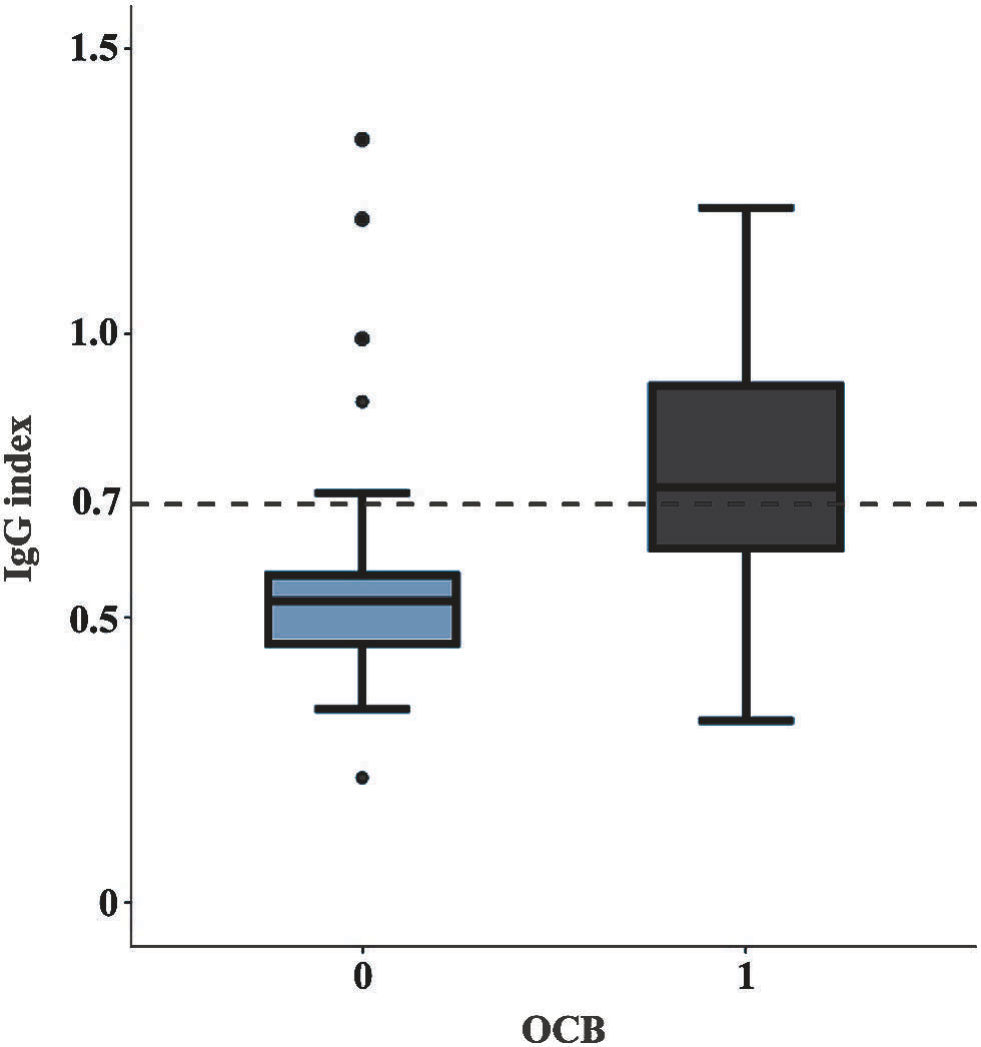
Correlation between OCB positivity and IgG index. In box-and-whisker plots the central horizontal bar shows the median IgG index, and the lower and upper boundaries show the 25th and 75th percentiles, respectively. The dashed horizontal line represents an IgG index of 0.7. IgG index positivity (IgG index > 0.7, the cut-off in our center and also the often-used cut-off in many other centers) was highly associated with the OCB status (Chi-square test, *X*^2^ = 22.90, *P* < 0.001). IgG, Immunoglobulin G; OCB, oligoclonal band.

Of the 105 patients, 44 (41.9%) has an elevated IgG index and 61 (58.1%) exhibited positive OCB at baseline (Table 1). Younger patients tended to have an elevated IgG index (student *t*-test, *P* = 0.004) and a positive OCB (student *t*-test, *P* = 0.005). Neither IgG index elevation nor OCB positivity was correlated with sex, presenting phenotype, presence of DIS at baseline, presence of spinal lesions at baseline, CSF cell count and CSF protein count (*P* > 0.05). Seventy-six patients were either IgG index-and-OCB double-positive or double-negative, leaving the remaining 26 having discordant results of IgG index and OCB. Notably, IgG index > 0.7 was highly associated with OCB positivity (*X*^2^ = 22.90, *P* < 0.001) (Figure 1). IgG index has a sensitivity of 62.3%, specificity of 86.4%, PPV of 86.4%, NPV of 62.3%, PLR of 4.57, and NLR of 0.44 for OCB positivity.

### Diagnostic Value of IgG Index vs. OCB

We first evaluated the diagnostic performance of IgG index alone in diagnosing CDMS among patients with CIS. Patients followed up for at least 2 years or having a CDMS conversion within 2 years were included in this analysis (*n* = 99). IgG index had a sensitivity of 51.47%, a specificity of 70.97%, PPV of 79.55%, NPV of 40.00%, PLR of 1.77, NLR of 0.68, and accuracy of 57.58%. By contrast, OCB displayed a significantly higher sensitivity of 67.65% (*P* < 0.001) and a lower specificity of 58.06% (*P* < 0.001). Both PLR (1.61) and NLR (0.56) were lower in OCB. PPV (77.97%), NPV (45.00%) and accuracy (64.65%) were similar between the 2 parameters (Table 2). However, neither an elevated IgG index nor OCB positivity was able to predict an earlier CDMS conversion, according to multivariate Cox analysis after adjusting for sex, DMT use and age of onset [adjusted hazard ratio [HR] = 1.36 [0.82–2.25], *P* = 0.23 for IgG index; adjusted HR = 1.55 [0.90–2.68], *P* = 0.11 for OCB].

**Table 2:** Diagnostic performance of IgG index and OCB.

***n* = 99^**#**^**	**Sensitivity**	**Specificity**	**PPV**	**NPV**	**PLR**	**NLR**	**Accuracy**
	**(95% CI)**	**(95% CI)**	**(95% CI)**	**(95% CI)**	**(95% CI)**	**(95% CI)**	**(95% CI)**
IgG index	0.51 (0.51–0.53)	0.71 (0.70–0.73)	0.80 (0.78–0.81)	0.40 (0.39–0.41)	1.77 (1.86–2.24)	0.68 (0.68–0.73)	0.58 (0.57–0.59)
OCB	0.68 (0.66–0.69)	0.58 (0.57–0.60)	0.78 (0.77–0.79)	0.45 (0.44–0.47)	1.61 (1.57–1.75)	0.56 (0.57–0.63)	0.65 (0.63–0.66)

# *Patients followed up for at least 2 years or having the clinically definite multiple sclerosis conversion within 2 years were included in this analysis*.

*CI, confidence interval; IgG, immunoglobulin G; PLR, positive likelihood ratio; NLR, negative likelihood ratio; NPV, negative predictive value; OCB, oligoclonal band; PPV, positive predictive value*.

We also evaluated the diagnostic performance of IgG index or OCB using McDonald MS as the outcome. Similarly, IgG index was less sensitive but more specific vs. OCB for McDonald MS (Supplementary Table 1). Both were not indicative of time to McDonald MS according to Cox analysis [adjusted hazard ratio [HR] = 1.35 [0.84-2.18] *P* = 0.21 for IgG index; adjusted HR = 1.43 [0.86–2.38] *P* = 0.16 for OCB].

### Prognostic Value of IgG Index vs. OCB

Next, we examined the prognostic value of IgG index for early disease activity and progression in the first 2 years. Patients followed up for at least 1 year (*n* = 97) or 2 years (n = 77) were included in the 1-year and 2-years analysis, respectively. Interestingly, an elevated IgG index was predictive of more clinical relapses both in the first [adjusted odds ratio [OR] = 1.32 (1.06–1.63), *P* = 0.015] and second year after onset [adjusted OR = 1.69 [1.13–2.52], *P* = 0.013]. In addition, we also found IgG index highly associated with EDSS worsening in the first [adjusted OR = 1.76 (1.03–3.01), *P* = 0.040] and second year [adjusted OR = 1.85 (1.07–3.22), *P* = 0.032] after onset. By contrast, OCB positivity at baseline failed to show a correlation with the number of clinical relapses and EDSS worsening in the first 2 years (*P* > 0.05). Both IgG index and OCB were uncorrelated with MRI activity (both cMRI and sMRI) in the first 2 years (*P* > 0.05) (Supplementary Table 2).

### Incorporating IgG Index Into the 2017 McDonald Criteria

Patients followed up for at least 2 years or having a CDMS conversion within 2 years were included in this analysis (*n* = 99). The modified criteria 1 (IgG index or OCB) displayed a similar sensitivity (74.55 vs. 69.09%, *P* = 0.25) and specificity (48.28 vs. 55.17%, *P* = 0.5) when compared with the original 2017 criteria; The modified criteria 2 (IgG index and OCB) yet displayed a significantly lower sensitivity (43.64 vs. 69.09%, *P* = 0.0001) but a significantly higher specificity (79.31 vs. 55.17%, *P* = 0.0015) than the 2017 criteria; The modified 3 (IgG index instead of OCB) displayed a similar sensitivity (58.18 vs. 69.09%, *P* = 0.146) and specificity (72.41 vs. 55.17%„ *P* = 0.180) than the 2017 criteria. Notably, the modified criteria 2 and 3 displayed the highest positive likelihood ratio (PLR) of 2.11 (vs. 1.54 of the 2017 criteria), whereas the modified criteria 2 showed the highest NLR ratio of 0.71(vs. 0.56 of the 2017 criteria) (Table 3). Cox analysis revealed that the modified criteria 2 [adjusted HR = 2.30 [1.27–4.15], *P* = 0.006] were highly predictive of an earlier CDMS conversion, after adjustment for sex, DMT use and age of onset.

**Table 3:** Diagnostic performance of the modified and 2017 McDonald criteria.

***n* = 99^**#**^**	**Sensitivity**	**Specificity**	**PPV**	**NPV**	**PLR**	**NLR**	**Accuracy**
	**(95% CI)**	**(95% CI)**	**(95% CI)**	**(95% CI)**	**(95% CI)**	**(95% CI)**	**(95% CI)**
2017 McDonald criteria	0.69 (0.68–0.70)	0.55 (0.54–0.57)	0.75 (0.73–0.75)	0.48 (0.48–0.51)	1.54 (1.51–1.58)	0.56 (0.56–0.59)	0.64 (0.64–0.65)
Modified criteria 1^##^	0.75 (0.74–0.76)	0.48 (0.46–0.49)	0.73 (0.72–0.74)	0.50 (0.48–0.52)	1.44 (1.44–1.49)	0.53 (0.52–0.54)	0.65 (0.65–0.66)
Modified criteria 2^###^	0.44 (0.42–0.45)	0.79 (0.78–0.80)	0.80 (0.78–0.80)	0.43 (0.42–0.44)	2.11 (2.08–2.23)	0.71 (0.70–0.72)	0.56 (0.55–0.57)
Modified criteria 3^####^	0.58 (0.57–0.60)	0.72 (0.71–0.74)	0.80 (0.79–0.81)	0.48 (0.46–0.49)	2.11 (2.07–2.22)	0.58 (0.57–0.59)	0.63 (0.62–0.64)

# *Patients followed up for at least 2 years or having the clinically definite multiple sclerosis conversion within 2 years were included in this analysis*.

## *The 2017 McDonald criteria was modified with replacement of OCB positivity by “IgG index or OCB positivity”*.

### *The 2017 McDonald criteria was modified with replacement of OCB positivity by “IgG index and OCB positivity”*.

#### *The 2017 McDonald criteria was modified with replacement of OCB positivity by “IgG index”*.

*CI, confidence interval; IgG, immunoglobulin G; PLR, positive likelihood ratio; NLR, negative likelihood ratio; NPV, negative predictive value; OCB, oligoclonal band; PPV, positive predictive value*.

We also evaluated the diagnostic performance of each criteria using McDonald MS as the outcome (Supplementary Table 3). Similarly, only the modified criteria 2 was significantly less sensitive IgG index was less sensitive (*P* < 0.001) but more specific (*P* = 0.016) than the 2017 criteria. According to multivariate Cox analysis, the modified criteria 2 and modified criteria 3 were highly predictive of an earlier conversion to McDonald MS [adjusted HR = 2.17 [1.24–3.79] *P* = 0.01 for modified criteria 2; adjusted HR = 1.87 [1.06–3.28] *P* = 0.03 for modified criteria 3].

## Discussion

Early diagnosis and treatment of MS remains a universal challenge and particularly a concern in Asia. CSF analysis, with its ability to detect intrathecal inflammation, is a valuable tool in this regard (3, 27). As a routinely performed workup for patients suspected of MS, IgG index is a reachable and cost-effective candidate as evidence of DIT (1). Its diagnostic and prognostic value yet lacks real-world evidence from Asian countries, where OCB result is often lacking at initial presentation, impeding an early diagnosis of MS (7, 28–30). In a cohort of CIS patients in China, we found that [1] IgG index > 0.7 is highly suggestive of OCB positivity; [2] IgG index has a higher specificity and PPV than OCB for MS diagnosis, and is indicative of early disease activity; [3] When OCB result is lacking at baseline, IgG index serves as a specific surrogate of OCB in the 2017 McDonald criteria; when OCB result is available, IgG index and OCB double-positivity strongly suggests the diagnosis of MS; whereas IgG index/OCB positivity facilitates the MS diagnosis without impairing the diagnostic performance.

Intrathecal immunoglobulin synthesis remains a crucial biological feature in MS, as entailed quantitatively by IgG index and qualitatively by OCB (1). OCB was known to be more sensitive in patients with MS than IgG index (90–100 vs. 50–75%) (3, 31). Its use for the early diagnosis of MS in China is yet limited by an undetermined specificity among Asians and the costly and time-consuming testing procedure (13, 15, 16). With the increasing recognition of the importance of early diagnosis and treatment in MS, the need for a more accessible marker as OCB surrogate is also becoming evident (2, 32). In our study, IgG index and OCB are highly correlated, with IgG index > 0.7 highly suggestive of OCB positivity (PPV: 86.4%, PLR 4.57). In previous studies in the Caucasian population, the correlation can be more significant with a PPV even reaching 99% with a cutoff of 0.7 and 96% with a cutoff of 0.8 (7, 8, 33). It should also be noted that IgG index ≤0.7 may not exclude a negative OCB (NPV: 62.3%, NLR 0.44), in line with previous studies (7, 33). This might be attributed to the inherent low sensitivity (52%) of the test given its methodology. IgG index measures the blood-CSF IgG transfer with the correction of CSF-serum albumin quotient by assuming a linear relationship between the *Q*_*alb*_ and the *Q*_*IgG*_ index. It is thereby prone to false-negatives, since *Q*_*alb*_ values can be affected by multiple factors including age, ethnicity and other environmental conditions (30, 34).

The diagnostic value of IgG index lies in its high PPV (80%), high PLR (1.77) and high specificity (72%) for CDMS conversion, compared with a lower PPV (78%), PLR (1.61) and specificity (58%) of OCB. This means that an elevated IgG index indicates a greater risk for developing MS in CIS patients in our cohort, even greater than that of OCB. A normal IgG index, however, does not equate to excluding the diagnosis. This result was echoed by a previous study on 460 patients from Austria (33). The lower diagnostic specificity of OCB for MS in our study, however, might be explained by the lower prevalence of both MS and OCB in China (16, 17, 35). Therefore, one potential implication for Asian patients is the utility of an elevated IgG index for MS diagnosis and the value of a negative OCB for the exclusion of the diagnosis (34). Taken together, the advantages of easy accessibility, lower cost, and a high diagnostic value of IgG index elevation prompted us to further examine its prognostic utility.

We found IgG index > 0.7 at baseline predictive of early inflammatory activity in the first 2 years as shown by clinical relapses and EDSS worsening. By contrast, OCB positivity failed to predict early disease activity in our study. This finding favoring the prognostic value of quantitative IgG synthesis over OCB was also indicated by several large studies before (10, 36–39). A recent prospective study with 1,376 German patients found that patients with an elevated IgG index were twice more likely to develop disability worsening 4 years after onset, whereas the presence of OCB failed to show a higher risk of EDSS worsening (10). Klein et al. also found IgG index elevation one of the strongest predictors of cMRI activity 1 year after onset in a cohort of 149 CIS patients, with OCB positivity showing a predictive value though with a smaller odds ratio (37). However, two large studies based on patients with an established diagnosis of MS revealed no association between either OCB status or quantitative measurement of IgG synthesis and disease progression in patients (13, 39). The discrepancy, however, was likely due to the inclusion of patients with long disease duration in the latter 2 studies. Altogether, our results suggested a higher predictive value of IgG index > 0.7 at baseline for early disease activity than OCB positivity, which may reflect the superior prognostic value of quantitative IgG measurement than the qualitative analysis. It should be noted that the measurement of intrathecal IgG synthesis is prone to influences from external factors, including the methodology of testing (4), age of onset (17), latitude (16), etc. Therefore, further confirmation would be needed to extrapolate our results in different populations.

**Figure 2:**
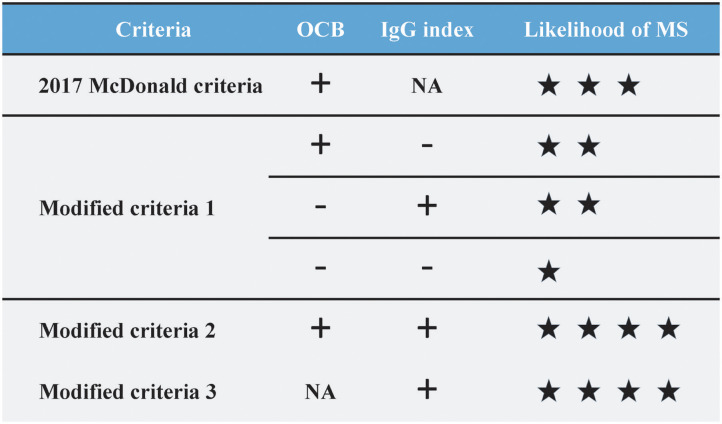
The likelihood of multiple sclerosis diagnosis based on OCB status and/or IgG index positivity in the 2017 McDonald criteria. When compared to the 2017 McDonald criteria (PLR 1.54, NLR 0.56), the modified criteria 2 (IgG index and OCB) and modified criteira 3 (IgG index) displayed a higher PLR (both 2.11) yet a higher NLR (0.71 and 0.58, respectively). The modified criteria 1 (IgG index or OCB) displayed a lower NLR (0.53) yet a lower PLR (1.44). IgG, Immunoglobulin G; OCB, oligoclonal band; PLR, Positive likelihood ratio; NLR, Negative likelihood ratio.

Making the correct diagnosis at the earliest time point remains the holy grail in MS (40). The 2017 McDonald criteria partly addressed the issue by including OCB as evidence of DIT. Yet the increase in sensitivity of the 2017 criteria was accompanied by a loss in specificity, according to validation studies from different populations (15, 41, 42). The limited accessibility and lower prevalence of OCB in Asia also interfere with early diagnosis and treatment (7, 17). To circumvent the issue, we attempted to incorporate IgG index into the 2017 McDonald criteria under different scenarios, hoping to make full use of the routinely performed test. Firstly, in cases when OCB result is lacking, we found that the use of IgG index as OCB surrogate (modified criteria 3) increases the specificity, PPV and PLR without impairing the NPV and accuracy. Secondly, in cases when OCB result is available, the presence of “IgG index and OCB double-positivity” (modified criteria 2) significantly increased the specificity, PPV and PLR of the diagnostic criteria yet at the expense of sensitivity, whereas “IgG index or OCB positivity” (modified criteria 1) displayed a slightly lower specificity, PLR with a higher sensitivity and lower NLR. LRs are one of the most practical metrics for efficient clinical diagnosis and decision-making (43). It is widely used for its incorporation of both sensitivity and specificity and independence of disease prevalence. Given the high PLR of the modified criteria 2 and 3, our findings support the diagnostic utility of an elevated IgG index as an OCB surrogate either in cases with an unknown OCB status or with OCB positivity. Alternatively, given the low NLR of the modified criteria 1, IgG index-and-OCB double-negativity is also valuable in excluding the MS diagnosis in the Asian population (Figure 2).

Our study had several limitations. Firstly, the small sample size may limit the power of our study. The inclusion of 105 patients in the final cohort is mainly due to the low prevalence of MS in China (14, 17, 28, 42), the lack of CSF data in some patients of CIS and the single center nature of our study. Several attempts were made to increase the validity of our results, including the standardized methodology of CSF analysis, the use of multivariate analysis and the employment of bootstrap method for more precise estimation of confidence intervals. The second is the use of 1.5T MRI in our center, which may underestimate the number of radiological relapses during follow-up. Given the limitation of MRI techniques, we thereby used other outcome measures in parallel for disease activity and progression (i.e., clinical relapses and EDSS worsening). Taken together, considering the potential clinical significance of IgG index for MS in the Asian population, future studies based on larger multicenter cohorts are needed to confirm our findings.

## Conclusion

In a cohort of CIS patients in China, we identified IgG index > 0.7 highly indicative of OCB positivity. An elevated IgG index at baseline was a specific marker for CDMS conversion, early disease activity and progression. IgG index, when elevated, could be harnessed as an OCB surrogate in the 2017 McDonald criteria in the Asian population, facilitating an earlier diagnosis of MS. Future studies with a larger cohort are needed to further validate our findings.

## Data Availability Statement

The raw data supporting the conclusions of this article will be made available by the authors, without undue reservation.

## Ethics Statement

The studies involving human participants were reviewed and approved by the ethics committee of the Second Affiliated Hospital School of Medicine Zhejiang University (approval number: 2019-082). All patients consented for the use of their anonymized MRI examinations and clinical details for research purposes.

## Author Contributions

YZ, M-TC, and Y-XZ contributed to the concept and design of the study. YZ and M-TC contributed to drafting the initial manuscript. FY, J-PZ, WF, and C-HS were responsible for reading the manuscript for intellectual content. Y-XZ and M-PD contributed to revising the manuscript for intellectual content. All authors approved the final version of the manuscript and contributed to the acquisition and analysis of the data. All authors contributed to the article and approved the submitted version.

## Conflict of Interest

The authors declare that the research was conducted in the absence of any commercial or financial relationships that could be construed as a potential conflict of interest.
