# Supplementary material for: IgG Index Revisited: Diagnostic Utility and Prognostic Value in Multiple Sclerosis
Source: Front Immunol. 2020 Aug 20;11:1799. doi: 10.3389/fimmu.2020.01799 (PMC7468492; doi:10.3389/fimmu.2020.01799)
Supplement: Supplementary file 1 [file Data_Sheet_1.docx]

**Supplementary Table 1**. Diagnostic performance of IgG index / OCB using McDonald MS as outcome.

| n = 99 | Sensitivity (95% CI) | Specificity (95% CI) | PPV  (95% CI) | NPV  (95% CI) | PLR (95% CI) | NLR (95% CI) | Accuracy  (95% CI) |
| --- | --- | --- | --- | --- | --- | --- | --- |
| IgG index | 0.51 (0.50-0.51) | 0.81 (0.78-0.82) | 0.91 (0.90-0.91) | 0.31 (0.30-0.32) | 2.69 | 0.60 (0.59-0.63) | 0.58 (0.56-0.58) |
| OCB | 0.65 (0.65-0.67) | 0.62 (0.60-0.64) | 0.86 (0.86-0.87) | 0.33 (0.31-0.34) | 1.72 (1.67-1.82) | 0.56 (0.56-0.59) | 0.65 (0.64-0.66) |

^#^ Patients followed up for at least 2 years or having the clinically definite multiple sclerosis conversion within 2 years were included in this analysis.

Abbreviations: CI, confidence interval; IgG, immunoglobulin G; PLR, positive likelihood ratio; NLR, negative likelihood ratio; NPV, negative predictive value; OCB, oligoclonal band; PPV, positive predictive value.

**Supplementary Table 2**. IgG index / OCB and early disease activity and progression.

|  | n | Parameters | Adjusted OR (95% CI) | *P* value |
| --- | --- | --- | --- | --- |
| 1-year analysis^#^ | | | | |
| IgG index | 97 | Clinical relapses | 1.32 (1.06-1.63) | 0.015* |
|  | 97 | Cranial MRI activity | 1.06 (0.68-1.63) | 0.806 |
|  | 51 | Spinal cord MRI activity | 1.84 (0.44-8.23) | 0.404 |
|  | 97 | EDSS worsening | 1.76 (1.03-3.01) | 0.040* |
| OCB | 97 | Clinical relapses | 1.12 (0.90-1.40) | 0.320 |
|  | 97 | Cranial MRI activity | 1.01 (0.66-1.58) | 0.953 |
|  | 51 | Spinal cord MRI activity | 1.47 (0.35-7.61) | 0.612 |
|  | 97 | EDSS worsening | 1.39 (0.81-2.38) | 0.241 |
| 2-year analysis^##^ | | | | |
| IgG index | 77 | Clinical relapses | 1.69 (1.13-2.52) | 0.013* |
|  | 77 | Cranial MRI activity | 1.16 (0.66-2.01) | 0.593 |
|  | 39 | Spinal cord MRI activity | 3.09 (0.48-27.75) | 0.255 |
|  | 77 | EDSS worsening | 1.85 (1.07-3.22) | 0.032* |
| OCB | 77 | Clinical relapses | 1.05 (0.69-1.60) | 0.802 |
|  | 77 | Cranial MRI activity | 1.07 (0.63-1.83) | 0.810 |
|  | 39 | Spinal cord MRI activity | 2.90 (0.62-17.9) | 0.304 |
|  | 77 | EDSS worsening | 1.59 (0.91-2.78) | 0.111 |

Significance codes:  * p<0.05;

^#^ Patients followed up for at least 1 year were included in the 1-year analysis;

^##^ Patients followed up for at least 2 years were included in the 2-year analysis.

Abbreviations: CI, confidence interval; EDSS, Expanded Disability Status Scale; IgG, immunoglobulin G; MRI, magnetic resonance imaging; OCB, oligoclonal band; OR, odds ratio.

**Supplementary Table 3.** Diagnostic performance of the 2017 McDonald criteria and the modified criteria using McDonald MS as outcome.

| n = 99^#^ | Sensitivity (95% CI) | Specificity (95% CI) | PPV  (95% CI) | NPV  (95% CI) | PLR (95% CI) | NLR (95% CI) | Accuracy  (95% CI) |
| --- | --- | --- | --- | --- | --- | --- | --- |
| 2017 McDonald criteria | 0.65 (0.64-0.66) | 0.52 (0.51-0.55) | 0.80 (0.79-0.81) | 0.33 (0.32-0.35) | 1.37 (1.34-1.44) | 0.67 (0.66-0.72) | 0.62 (0.61-0.63) |
| Modified criteria 1^##^ | 0.71 (0.70-0.72) | 0.48 (0.47-0.50) | 0.80 (0.80-0.81) | 0.36 (0.35-0.37) | 1.36 (1.39-1.52) | 0.60 (0.58-0.64) | 0.65 (0.65-0.66) |
| Modified criteria 2^###^ | 0.43 (0.42-0.44) p<0.001 | 0.86 (0.85-0.87) p=0.016 | 0.90 (0.89-0.91) | 0.33 (0.33-0.35) | 3 | 0.67 (0.66-0.69) | 0.54 (0.53-0.55) |
| Modified criteria 3 | 0.57 (0.57-0.59) | 0.81 (0.79-0.83) | 0.90 (0.89-0.91) | 0.39 (0.37-0.40) | 3 | 0.53 (0.53-0.56) | 0.63 (0.63-0.65) |

^#^ Patients followed up for at least 2 years or having the clinically definite multiple sclerosis conversion within 2 years were included in this analysis (n = 99).

^##^ The 2017 McDonald criteria was modified with replacement of OCB positivity by “IgG index or OCB positivity”.

^###^ The 2017 McDonald criteria was modified with replacement of OCB positivity by “IgG index and OCB positivity”.

^####^ The 2017 McDonald criteria was modified with replacement of OCB positivity by “IgG index”.

Abbreviations: CI, confidence interval; IgG, immunoglobulin G; PLR, positive likelihood ratio; NLR, negative likelihood ratio; NPV, negative predictive value; OCB, oligoclonal band; PPV, positive predictive value.
